# Advance care planning engagement in patients with chronic, life-limiting illness: baseline findings from a cluster-randomised controlled trial in primary care

**DOI:** 10.3399/BJGP.2022.0100

**Published:** 2023-04-04

**Authors:** Julie Stevens, Rose Miranda, Luc Deliens, Peter Pype, Aline De Vleminck, Koen Pardon

**Affiliations:** End-of-Life Care Research Group, Vrije Universiteit Brussel (VUB) and Universiteit Gent; Department of Family Medicine and Chronic Care, VUB, Brussels; Department of Public Health and Primary Care, Universiteit Gent, Ghent.; End-of-Life Care Research Group, VUB and Universiteit Gent; Department of Family Medicine and Chronic Care, VUB, Brussels.; End-of-Life Care Research Group, Vrije Universiteit Brussel (VUB) and Universiteit Gent; Department of Family Medicine and Chronic Care, VUB, Brussels; Department of Public Health and Primary Care, Universiteit Gent, Ghent.; End-of-Life Care Research Group, VUB, and Universiteit Gent; Department of Public Health and Primary Care, Universiteit Gent, Ghent.; End-of-Life Care Research Group, VUB and Universiteit Gent; Department of Family Medicine and Chronic Care, VUB, Brussels.; End-of-Life Care Research Group, VUB and Universiteit Gent; Department of Family Medicine and Chronic Care, VUB, Brussels.

**Keywords:** advance care planning, baseline survey, chronic disease, general practice, patient participation, surveys and questionnaires

## Abstract

**Background:**

Advance care planning (ACP) has been characterised as a complex process of communication and decision making. For ACP behaviour change, underlying processes such as self-efficacy and readiness are needed. However, studies about which patient characteristics are associated with ACP have mainly focused on whether ACP actions are completed, leaving behaviour change processes unexplored.

**Aim:**

To assess whether patients’ characteristics and patient-perceived quality of GP ACP communication were associated with patients’ ACP engagement.

**Design and setting:**

Baseline data were used from the ACP-GP cluster-randomised controlled trial in patients with chronic, life-limiting illness (*n* = 95).

**Method:**

Patients completed questionnaires detailing demographic and clinical characteristics, and their perception about their GPs’ ACP information provision and listening. Engagement was measured using the 15-item ACP Engagement Survey, with self-efficacy and readiness subscales. Linear mixed models tested associations with engagement.

**Results:**

Demographic and clinical characteristics were not associated with engagement; nor was how much ACP information patients received from their GP or the extent to which the GP listened to what was important for the patient to live well or important to the patient regarding future care. Higher overall ACP engagement (*P* = 0.002) and self-efficacy (*P*<0.001) were observed in patients who gave a high rating for the extent to which their GP listened to their worries regarding future health.

**Conclusion:**

This study suggests that GPs providing information about ACP alone is not associated with a patient’s ACP engagement; an important element is to listen to patients’ worries regarding their future health.

## INTRODUCTION

Patients with chronic, life-limiting illness often still receive medical care that does not align with their values and preferences.^1^ Advance care planning (ACP) can reduce this discrepancy by promoting communication and understanding of patients’ values and preferences for future (end-of-life) care before loss of decisional capacity.^2^ ACP is a complex process of communication and decision making, which includes actions such as contemplating care wishes, having conversations about values and care preferences with family and health providers, completing advance directives for future care, and revisiting these actions over time.^3^ Although studies show that adults in the community as well as patients think about and are open to ACP,^4^^,^^5^ conversations and corresponding documentation remain infrequent.^6^^–^9 This has also been found in Belgium, where the prevalence of advance directives to withhold or withdraw treatment is low for patients who are terminally ill.^10^ For patients with cancer specifically, GPs in Belgium are aware of patient preferences for treatment at the end of life in approximately one-half of cases, and of patient preferences for a surrogate decision maker in less than one-third of cases.^11^

Evidence from the literature about which personal characteristics are associated with ACP engagement has mainly focused on whether ACP actions are performed. Increasing age has been found not only to be associated with increased likelihood of having ACP documentation^12^^–^15 but also with a decreased likelihood of discussing ACP with family and friends.^16^ Female sex has been found to be associated with having discussions about end-of-life care wishes,^6^^,^^8^^,^^17^ but findings regarding completion of ACP documents are mixed.^12^^,^^14^ Examples of other factors that may correlate with ACP actions include religious beliefs and religiosity,^8^^,^^14^^,^^17^^–^19 educational attainment,^8^^,^^13^^,^^14^^,^^17^^,^^20^ marital status,^19^^,^^21^ and physical functioning.^8^^,^^12^^,^^18^^,^^22^

In comparison, studies that examine ACP as a behaviour change process, instead of discrete actions as described above, are fewer. Behaviour change theory and social cognitive theory have been used to describe processes underlying ACP engagement, including self-efficacy (that is, how confident the patient feels to complete the behaviour) and readiness (that is, the patient’s stage of behaviour change).^16^^,^^23^ Based on these theoretical foundations, the ACP Engagement Survey has been developed to measure behaviour change processes (knowledge, contemplation, self-efficacy, and readiness) and actions (for example, whether discussions have occurred).^23^ Using this survey it has been found that patients with depression or anxiety have higher engagement.^24^ In a validation of the Dutch 34-item ACP Engagement Survey, patients aged ≥60 years and with chronic disease showed higher engagement;^22^ however, the study did not compare engagement within the chronic illness category (cancer and non-cancer diseases). To the authors’ knowledge, no other studies have examined how other demographic and clinical characteristics relate to ACP as a process of behaviour change.

**Table:** How this fits in

Although changes to advance care planning (ACP) behaviour may require changes to underlying processes of behaviour change, the factors associated with these processes are largely unexplored. This study found that patients’ demographic and clinical characteristics were not associated with their ACP engagement, a measure consisting of both self-efficacy and readiness for ACP. However, patients who felt that their GP listened to what their worries were regarding their future health had higher overall engagement and higher self-efficacy. This supports the importance of active listening when discussing ACP with patients, and especially of paying attention to the worries patients may have about the impact of future health states.

As ACP is a process of communication, factors pertaining to how GPs, who play a pivotal role in initiating ACP because of their accessibility and continuity of care,^5^^,^^25^ communicate with the patient should also be considered. GPs’ communication skills, including active listening and a positive attitude towards ACP, have been described as enablers of ACP uptake;^26^^,^^27^ however, whether the patients’ perceptions of GPs’ communication relate to the patients’ ACP engagement has not yet been explored.

Examining the impact of these factors on behaviour change processes for ACP can shed light on which determinants play a role in ACP behaviour change. This information can be taken into account when developing models of ACP for future interventions in the primary care setting. The purpose of this study was therefore to explore ACP engagement in a study population of patients with chronic, life-limiting illness, and to understand the association between patients’ ACP engagement and their demographic and clinical characteristics, and their perceived extent of ACP-related communication with their GP.

## METHOD

### Setting

This survey study used the baseline data from a cluster-randomised controlled trial (RCT) aiming to evaluate an ACP intervention in general practice (ACP-GP).^28^ As this intervention involves the training of GPs, patients were clustered by GP practice.

### Participants

In total, 35 Dutch-speaking GPs working in Flanders or Brussels, Belgium, were recruited for the purpose of the RCT. Recruited GPs identified eligible patients. Inclusion criteria for patients were Dutch-speaking adults (aged >18 years) with a chronic, life-limiting illness (cancer, organ failure, and/or frailty) for whom the GP answered ‘no’ to the question: ‘Would I be surprised if this patient were to die within the next 12 to 24 months?’

Patients with cognitive impairment; who were unable to provide consent or complete the questionnaires; for whom the GP would not be surprised if they were to die within the next 6 months; and who had participated in the pilot study of the intervention or were participating in similar studies were excluded.

### Data-collection procedures

Data collectors approached patients for written informed consent and questionnaire completion. When COVID-19 restrictions prohibited home visits, informed consent and questionnaires were collected via postal mail combined with telephone contact by the data collectors. Baseline data were collected from October to December 2020.

### Measurements

Patients’ demographic data included age, sex, marital status, education, religion, the person most involved in the patient’s care, and whether this person lives with the patient. For clinical characteristics, the severity of anxiety symptoms were measured using the seven-item General Anxiety Disorder (GAD-7) scale^29^ and the severity of depression symptoms with the nine-item Patient Health Questionnaire (PHQ-9).^30^ Both scales are sums of Likert items, where higher scores indicate greater symptom severity. Health-related quality of life was measured with the Short-Form Health Survey (SF-12v2),^31^ which yields summary measures for physical and mental health (mean 50, standard deviation [SD] 10). Scores range from 0 to 100, higher scores indicating better health.^31^

Diagnosis was not included in the patient questionnaire but was ascertained by the data collectors and checked with the GP if there was uncertainty.

For patients’ perception of the quality of ACP communication with their GP, on a 10-point Likert scale patients indicated how much information they received from their GP about ACP; and to what extent their GP listened to what is important for them to live well, what is important to them regarding future care (for example, place of care), and their worries regarding future health (for example, pain and/or illness exacerbation).

ACP engagement was measured using the 15-item version of the ACP Engagement Survey, which has been validated with patients with chronic medical illness and can be used to detect differences in ACP behaviour processes.^23^^,^^32^ The 15-item version was selected as it reduces response burden while retaining two crucial subscales for ACP engagement, that is, self-efficacy and readiness, across four ACP domains: surrogate decision makers, values and quality of life, flexibility in surrogate decision making, and asking doctors questions. Items are on a five-point Likert scale, with higher scores indicating higher engagement. The English version of the survey underwent forward–backward translation and cognitive testing with six patients, who met the same inclusion criteria as those described above.

### Statistical analyses

Descriptive statistics were used to describe patient characteristics and quality of patient-perceived ACP communication from their GP. As responses were not normally distributed for patient-perceived ACP communication by the GP, in this study these scales were divided into categories: ‘low rating’ (points 1–5) and ‘high rating’ (points 6–10).

Scale scores (ACP Engagement Survey total and subscales, GAD-7, PHQ-9, and SF-12v2) were calculated for patients with <25% missing values on a given scale. When >25% of responses were missing for a given scale, the scale score was coded as missing. For the GAD-7 and PHQ-9 the sum was rescaled by dividing by the proportion of valid items. No item-level missingness was allowed for the SF-12v2, as the summary scores were computed through aggregating and weighting. When missingness was <25% for this scale, missing values were estimated using the expectation-maximalisation procedure,^33^ with all valid items and the responder’s age used for estimation.

The sample means for ACP engagement total score and the two subscales were calculated. To account for clustering within GPs, linear mixed models were used to analyse the associations between patient engagement and their characteristics, and quality of patient-rated GP ACP communication. All association analyses were adjusted for multiple testing using the Benjamini–Hochberg procedure, false discovery rate set to 5%. Analyses were performed in IBM SPSS Statistics (version 27). Crude *P*-values are reported and ones that remained significant after adjustment are highlighted.

## RESULTS

The 35 recruited GPs identified 95 patients who gave informed consent and returned questionnaires.

About half of these 95 patients were aged ≥80 years (50.5%, *n* = 48), female (52.6%, *n* = 50), and married, in a civil union, or a domestic partnership (47.4%, *n* = 45) (Table 1). Most patients (65.3%, *n* = 62) had completed education up to secondary school.

**Table 1.** Participant demographic and clinical characteristics (*N* = 95)

**Characteristic**	***n* (%)^a^**
**Age, years**	
<80	47 (49.5)
≥80	48 (50.5)

**Sex, female**	50 (52.6)

**Marital status**	
Married, civil union, or domestic partnership	45 (47.4)
Widow(er)	37 (38.9)
Divorced or single, never married	13 (13.7)

**Highest education attained**	
Primary school	18 (18.9)
Secondary school	62 (65.3)
Post-secondary school	13 (13.7)
None of the above	2 (2.1)

**Person most involved in care^b^**	
Spouse or partner	35 (37.2)
Child	32 (34.0)
Other family member	12 (12.8)
Other, not family member	13 (13.8)
No person identified	2 (2.1)

**Living together with person most involved in care^c^**	35 (37.6)
**Religion**	
Religious (Christianity)	57 (60.0)
Not religious	35 (36.8)
Prefer not to say	3 (3.2)

**Diagnosis**	
Cancer	32 (33.7)
Non-cancer	63 (66.3)

**Health-related quality of life (SF-12v2),^d^ mean (SD)**	
Physical health score	37.25 (11.02)
Mental health score	48.84 (12.49)

**Anxiety symptom severity (GAD-7),^e^ mean (SD)**	4.88 (4.49)
**Depressive symptom severity (PHQ-9),^f^ mean (SD)**	5.32 (4.38)

a *Unless otherwise stated.*

b *Missing*, n *= 1*

c *Missing*, n *= 2*.

d *Norm-based (mean 50, SD 10) score based on 1998 general US population means and SDs, range 0–100 with higher scores indicating better health.*

e *Sum ranging from 0 to 21. Higher scores indicate greater anxiety symptom severity. 0–4: minimal symptoms. 5–9: mild symptoms. 10–14: moderate symptoms. 15–21: severe symptoms.*

f *Sum ranging from 0 to 27. Higher scores indicate higher depressive symptom severity. 0–4: minimal symptoms. 5–9: mild symptoms. 10–14: moderate symptoms. 15–19: moderately severe symptoms. 20–27: severe symptoms. GAD-7 = seven-item General Anxiety Disorder. PHQ-9 = nine-item Patient Health Questionnaire. SD = standard deviation. SF-12v2 = Short-Form Health Survey.*

For 37.2% (*n* = 35/94) of patients, their spouse or partner were most involved in their care; for 34.0% (*n* = 32/94) it was their child; and 37.6% (*n* = 35/93) of patients lived together with the person most involved in their care. Of the 60.0% (*n* = 57/95) who indicated being religious, all were Christian. One-third (33.7%, *n* = 32/95) had an active cancer diagnosis. The average physical health score was 37.25 (SD 11.02); the average mental health score was 48.84 (SD 12.49). The average symptom severity was minimal-to-mild for anxiety (mean 4.88, SD 4.49) and mild for depression (mean 5.32, SD 4.38).

Approximately one-third of patients gave a high rating to how much information they had received from the GP about ACP (36.4%, *n* = 32/88) (Table 2). More than three-fourths of patients rated the GP highly when they were asked to what extent their GP listened to what is important for them to live well (82.0%, *n* = 73/89), what is important for them regarding future care (78.2%, *n* = 68/87), and their worries for their future health (77.3%, *n* = 68/88).

**Table 2.** Patient-perceived quality of GP ACP communication (*N* = 95)

**Questions^a^**	**‘High rating’ response to the question, *n* (%)**
How much information have you received from your GP about ACP?^b^	32 (36.4)
To what extent did your GP listen to what is important for you to live well?^c^	73 (82.0)
To what extent did your GP listen to what is important to you regarding your future care?^d^	68 (78.2)
To what extent did your GP listen to what your worries are regarding your future health?^e^	68 (77.3)

a *Ratings based on a 10-point Likert scale ranging from ‘not at all’ to ‘very much’; high rating: 6–10, low rating: 1–5. Period: past 3 months. Two patients had not had a consultation with their GP in the past 3 months.*

b *Missing*, n *= 7.*

c *Missing*, n *= 6.*

d *Missing*, n *= 8.*

e *Missing*, n *= 7. ACP = advance care planning.*

The mean total ACP engagement score was 3.06 (SD 0.98) (Table 3); mean self-efficacy was 3.86 (SD 1.13), and mean readiness was 2.52 (SD 1.20). Patient demographic or clinical characteristics were not associated with ACP engagement (Supplementary Table S1). Higher total engagement was found for patients who gave a high rating to the extent to which their GP listened to their worries for future health (3.27 versus 2.48, *P* = 0.002), compared with patients who gave a low rating. The same pattern was observed for self-efficacy (4.10 versus 3.14, *P*<0.001). The remaining items pertaining to GP communication were not significantly associated with engagement.

**Table 3.** ACP engagement across study sample^a^

**Question**	**Mean (SD)**
**ACP Engagement Survey, total score^b^**	3.06 (0.98)

How confident are you that today, you could …	
Medical decision makers	
Ask someone to be your medical decision maker?	4.08 (1.40)
What matters most in life	
Talk with your decision maker about the care you would want if you were very sick or near the end of life?	3.85 (1.52)
Talk with your doctor about the care you would want if you were very sick or near the end of life?	3.96 (1.41)
Flexibility	
Talk with your medical decision maker about how much flexibility you want to give your medical decision maker?	3.55 (1.54)
Talk with your doctor about how much flexibility you want to give your medical decision maker?	3.70 (1.40)
Asking your doctor questions	
Ask the right questions of your doctor to help make good medical decisions?	4.02 (1.30)

**How ready are you to …^c^**	
Medical decision makers	
Formally ask someone to be your medical decision maker?	2.65 (1.74)
Talk with your doctor about who you want your medical decision maker to be?	2.57 (1.65)
Sign official papers naming a person or group of people to make medical decisions for you?	2.54 (1.56)
What matters most in life	
Talk to your decision maker about the kind of medical care you would want if you were very sick or near the end of life?	2.62 (1.58)
Talk to your doctor about the kind of medical care you would want if you were very sick or near the end of life?	2.68 (1.42)
Sign official papers putting your wishes about the kind of medical care you would want if you were very sick or near the end of life?	2.51 (1.49)
Flexibility	
Talk to your decision maker about how much flexibility you want to give them?	2.18 (1.44)
Talk to your doctor about how much flexibility you want to give your decision maker?	2.18 (1.28)
Asking your doctor questions	
Ask your doctor questions to help you make a good medical decision?	2.61 (1.53)

a *ACP self-efficacy range: 1 (not at all confident) to 5 (very confident). ACP readiness range: 1 (I have never thought about it) to 5 (I have already done it).*

b *Missing,* n *= 1.*

c *Missing*, n *= 3. ACP = advance care planning. SD = standard deviation.*

## DISCUSSION

### Summary

The aim in the current study was to explore whether patients’ ACP engagement was associated with their demographic and clinical characteristics, and their perception of the quality of ACP communication with their GP. Most patients gave their GP a high rating for the extent to which they listened to what is important to the patient to live well and in regards to future care, and to patients’ worries for their future health. Fewer patients rated highly the amount of information they received from their GP about ACP.

After correction for multiple comparisons, the study found that patients who gave a high rating for the extent to which the GP listened to their worries regarding their future health showed higher engagement overall as well as higher self-efficacy.

### Strengths and limitations

To the authors’ knowledge, this is the first study to examine ACP behaviour change processes using the ACP Engagement Survey in Belgium, as well as the first to examine its associations with GP communication. This validated instrument reflects behaviour change constructs for multiple components of ACP, which can provide insight beyond whether or not patients complete discrete actions. By exploring patient-related factors such as demographics and clinical characteristics, as well as patient perceptions of their GPs’ listening and information provision, the current study has further disentangled which factors are important in ACP engagement.

Several limitations should be considered. This was a cross-sectional baseline assessment of a fairly limited sample of GPs and patients recruited in the context of an RCT in the Flanders and Brussels regions in Belgium. The findings can therefore not be generalised to the population with chronic illness and may differ for adults who have not had personal experience with chronic, life-limiting illness. Nevertheless, the focus was to explore factors associated with ACP engagement in this sample with chronic, life-limiting illness, which was achieved. As a result of the cross-sectional design, causality cannot be inferred. It is possible that patients who are more confident also participate more actively in conversations, and thus perceive their GP as listening to their concerns. Additionally, an attempt was made to limit recall bias by restricting questions about GPs’ information provision and listening to the 3 months before baseline assessment. Overall missingness for these items was limited, with no question missing >10%. Although data were collected about the patients’ perceptions of the quality of their GPs’ ACP communication, for the baseline assessment data were not collected about the timing, duration, and specific content of the pre-baseline consultation(s) during which these topics were discussed. As there is no single standardised process for ACP conversations in Belgium these conversations may vary from patient to patient. It may be useful for future research to also explore which aspects of the consultation(s) contribute to the patients’ perceptions.

### Comparison with existing literature

Contrary to the associations between sociodemographic and clinical characteristics and ACP actions observed in previous studies,^11^^–^21 the current study showed no associations between patients’ characteristics and their ACP engagement. This is potentially owing to the current sample being older and comprised of patients with chronic, life-limiting illness. As patients may find ACP increasingly relevant as they age or their health deteriorates,^34^^–^38 this sample may already have experienced more triggers for ACP, such as diagnosis of a chronic condition. ACP engagement has also been found to be associated with anxiety and depression in patients,^24^ but this was not found in the current study.

Further, no significant association was found with how much information patients felt they had received from their GP about ACP. Providing information can help to clarify and answer patients’ questions,^39^ but conversations should also leave space for patients to express their concerns.^40^^,^^41^ In particular, significant associations were found for patients’ ratings of the extent to which their GP listened to what their worries are regarding future health with overall engagement and self-efficacy. It is possible that engagement in ACP in patients with a chronic, life-limiting illness comes from worries about the impact of future health states, such as the burden their illness places on loved ones.^42^^–^44 Discussing such worries during the consultation can provide the basis for discussions about ACP.^45^

In patients with cancer, although an attentive, empathic communication style has been shown to be associated with their self-efficacy to cope with disease and treatment,^46^ this study shows that communication is also associated with self-efficacy for ACP. Improving readiness, on the other hand, may require an approach tailored to the patient’s current stage of behaviour change; literature on stage-matching interventions exists^47^^,^^48^ but is still limited.

### Implications for research and practice

The current findings regarding the lack of associations between patients’ demographic or clinical characteristics and their ACP engagement support proactively offering ACP as standard to all patients with chronic, life-limiting illness, regardless of their sociodemographics, diagnosis, or functional status.^44^^,^^49^ This study also highlights that GPs providing information alone seems insufficient, and this should thus be combined with active listening to patients’ worries regarding their health.

Investigating the underlying behaviour change processes of self-efficacy and readiness yields important insights into which factors should be accounted for when creating models of ACP behaviour change processes. Considering the need to facilitate ACP in patients with chronic, life-limiting illness,^49^ this study emphasises the importance of active listening as a springboard in the ACP process. Formalising conversations from talking about worries about future health into actions such as discussing care at the end of life, talking to and appointing a surrogate decision maker, and documenting care wishes may be the next step in high-quality ACP in the general practice setting.^39^ Communication techniques such as these are already recommended as part of best-practice guidelines.^44^^,^^50^^,^^51^ The communication factor identified in this study can be attended to by GPs during conversations with their patients and may be amenable to change as investing in training can help practitioners further develop these skills.^45^^,^^52^^,^^53^ Importantly, these skills are also targeted in the ACP training intervention being delivered to GPs during the RCT, for which these baseline data were gathered.^28^

## Data

Data are available on reasonable request.
